# Speckle Contrast as Retinal Tissue Integrity Biomarker in Patients with Type 1 Diabetes Mellitus with No Retinopathy

**DOI:** 10.3390/jpm12111807

**Published:** 2022-11-01

**Authors:** Elvira Orduna-Hospital, Maria Arcas-Carbonell, Ana Sanchez-Cano, Isabel Pinilla, Alejandra Consejo

**Affiliations:** 1 Department of Applied Physics, University of Zaragoza, 50009 Zaragoza, Spain; 2 Aragon Institute for Health Research (IIS Aragon), 50009 Zaragoza, Spain; 3 Department of Ophthalmology, Lozano Blesa University Hospital, 50009 Zaragoza, Spain; 4 Department of Surgery, University of Zaragoza, 50009 Zaragoza, Spain

**Keywords:** retina, choroid, type 1 diabetes mellitus, speckle contrast, optical coherence tomography, inner retinal layer

## Abstract

Purpose: To study the retinal and choroidal layers in type 1 diabetes mellitus (DM1) without diabetic retinopathy (DR), using speckle contrast of optical coherence tomography (OCT) images as a tissue biomarker in comparison with healthy subjects. Methods: OCT Spectralis images of 148 eyes, 84 from DM1 patients without DR signs, and 64 belonging to the control group, were collected. The speckle contrast and thickness of the inner retinal layer (IRL), the outer retinal layer (ORL), and the choroidal layer in the nasal parafoveal area (N3), were prospectively analyzed. Results: A statistically significant difference (*p* = 0.001) in the IRL thickness between groups was observed, being thicker in the DM1 group. There were no differences in the ORL and choroidal thicknesses between groups. A statistically significant difference (*p* = 0.02) in the IRL speckle contrast was obtained, being lower in the DM1 group. The maximum speckle contrast was reached in the ORL for both groups, although in the DM1 group, it occurs closer to the choroid, at 64 ± 8 μm (*p* = 0.008). Conclusions: Statistically significant differences were found in speckle contrast and thickness between the control and the DM1 group, suggesting an IRL alteration of DM1 patients, supporting the retinal neurodegeneration before DR signs are observed.

## 1. Introduction

Diabetic retinopathy (DR) is one of the main causes of vision loss worldwide [1], especially in type 1 diabetes mellitus (DM1). In these patients, before the microvascular changes can be subjectively detected by an ophthalmologist in the eye fundus examination, functional changes occur, such as decreased visual acuity, contrast sensitivity, or color vision, due to the loss of retinal neurons, secondary to a neurodegenerative process [2,3,4,5,6]. Thanks to the new non-invasive imaging techniques developed, such as optical coherence tomography (OCT), it is possible to acquire multiple consecutive high-resolution images, distinguishing the different retinal layers and the choroid. To improve the choroidal visualization, the 840 nm wavelength spectral-domain (SD)-OCT with enhanced depth imaging (EDI) manages to invert the retinal image reaching the sensitivity to distinguish the choroid scleral limit. This technology allows the visualization, segmentation, and quantification of different retinal layers and choroidal thickness thanks to the refractive properties of each layer [7,8].

Changes in retinal macular thickness in diabetes prior to any sign of DR have been demonstrated [9,10,11], mainly affecting inner retinal layer (IRL) thickness, which is comprised from the innermost to outermost layer by retinal nerve fiber layer (RNFL), ganglion cell layer (GCL), and inner plexiform layer (IPL) [12,13]. In a retrospective study with a long-time follow-up with Spectralis SD-OCT, thinning of IRL thickness was observed in DM1 with disease progression before the appearance of DR signs [14]. Choroidal abnormalities have also been described in diabetic patients suffering from DR, showing a choroidal thickness decrease with proliferative DR or diabetic macular edema (DME) and few changes in patients with non-proliferative DR or without DR [15,16,17,18,19,20,21].

Even though alterations in retinal and choroidal thickness due to DM1 have been repeatedly reported [9,10,11,12,13,14,15,16,17,18,19,20,21], little is known about the intrinsic tissue changes occurring in the retinal and choroidal areas. The statistical analysis of OCT speckle could shed some light on retinal and choroidal tissue characterization. Speckle has historically been considered a source of noise in coherent light imaging, such as OCT. However, several works in OCT imaging have shown that speckle patterns may contain relevant information regarding the structural properties of the tissues from which it originated [22]. In ophthalmology, OCT speckle analysis has been already successfully applied to investigate cornea [23,24,25,26,27,28] and retinal vascularization [29]. Even though there exists ophthalmological evidence that highlights the potential of speckle analysis as a disease biomarker [26,27,29], further research is needed to understand the capabilities of speckle analysis as a disease biomarker in retinal research.

DM1 is a chronic disease with progressive retinal neuronal involvement. Consequently, retinal control is of paramount importance. The purpose of this study was to investigate the retinal and choroidal tissue of DM1 patients without DR. To do so, the speckle contrast of IRL, outer retinal layer (ORL), and choroid imaged by Spectralis SD-OCT was assessed in DM1 patients without DR and compared with an age-matched control group.

## 2. Materials and Methods

### 2.1. Suspects and Protocol

This retrospective study includes 84 eyes from 42 DM1 patients with no DR and 64 eyes from 32 age-matched healthy subjects [13]. No clinical data were collected specifically for this study. The study was carried out following the principles established in the Helsinki Declaration after the approval of the local Ethics Committee for Clinical Research of Aragon (CEICA 18/2017), and detailed consent forms were obtained from each participant.

DM1 patients were controlled by the endocrinology unit. Blood samples were analyzed every six months. Glycosylated hemoglobin (HbA1c), lipid values, and arterial blood pressure were maintained under extreme control, always within their respective range of normality.

The inclusion criteria for the DM1 group were DM1 diagnosis with no retinal changes identified by biomicroscopy or structural OCT; all subjects, the DM1 and the control groups had a best corrected visual acuity (BCVA) over 20/25 on the Snellen chart, with refractive errors between +5.00 to −5.00 diopters, normal anterior pole examination with slit-lamp and no fundoscopy anomalies. The control group included healthy sex- and age-matched subjects to the DM1 group.

Exclusion criteria for both groups were the presence of any sign of DR, glaucoma, or intraocular pressure (IOP) over 21 mmHg assessed by Goldmann applanation tonometry, optic nerve pathology, ocular inflammation, or previous ocular surgery or procedure including laser therapy, ocular trauma, anterior segment pathology or media opacification. Smokers were also excluded. At each patient’s visit, a detailed familiar, systemic, and ophthalmological medical history was performed.

The axial length (AL) was measured with the optical biometer IOLMaster 500 (Carl Zeiss Meditec, Oberkochen, Germany) and had to be between 20 and 28 mm to be included in the study.

**Figure 1:**
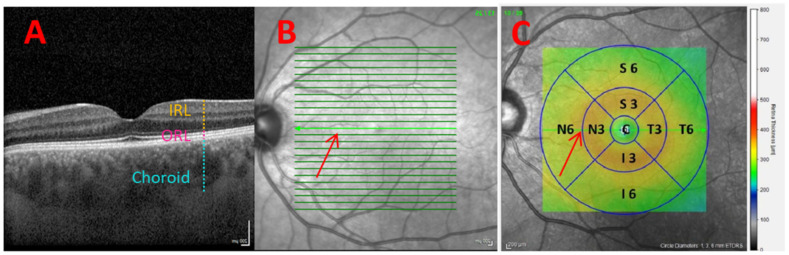
(**A**) Tomographic image of the central B-scan indicating inner retinal layer (IRL), outer retinal layer (ORL), and choroid. (**B**) 25 B-scans at 30° centered on the fovea, indicating the selected one with the red arrow. (**C**) The 9 Early Treatment Diabetic Retinopathy Study (ETDRS) areas for a left eye.

Each individual had both eyes imaged using a Spectralis SD-OCT (Heidelberg Engineering, Inc., Heidelberg, Germany) with the EDI volume fast macula scanning protocol, which performs 25 B-scans. The Spectralis SD-OCT images were performed always by the same observer between 1:00 p.m. and 4:00 p.m. The subject was asked to look into the internal fixation target, and Tru-Track eye-tracking technology was used. As indicated in Figure 1C Spectralis SD-OCT provides a circular macular map analysis, divided into nine sectorial thickness measurements into three concentric circles with diameters of 1, 3 (inner), and 6 (outer) mm forming the nine areas corresponding to the Early Treatment Diabetic Retinopathy Study (ETDRS) [30]. The Spectralis software version was 6.8.1.0. The quality of the scans was checked, and poor-quality and crooked scans were rejected. The threshold for image quality was at least 25 over 40 dB without artifacts. Retinal and choroidal thickness were assessed as described in previous work [13]. In short, the IRL was considered the space between the internal limiting membrane (ILM) and external limiting membrane (ELM), while the ORL was considered the space between the ELM and Bruch’s membrane (BM) using the Spectralis OCT built-in retinal layer segmentation software. Choroidal thickness was assessed manually with the support of the same OCT layer segmentation software.

The OCT image corresponding to the central B-scan (Figure 1A,B) was chosen to analyze speckle contrast. The speckle analysis was performed at the border between nasal parafoveal (N3) and perifoveal (N6) rings, thus being 1.5 mm from the fovea as indicated by the red arrow in Figure 1C. This point was chosen for the speckle analysis because it is just where there is a reasonable GCL thickness, located mostly in the parafoveal ring, and there is also a certain RNFL thickness, located mainly in the perifoveal ring. Both layers, the GCL and the RNFL are the ones that have been seen to undergo the greatest changes during the diabetes progress. The IRL, ORL, and choroidal thickness corresponding to the mean of the N3 and N6 were collected from previous work [13].

### 2.2. Data Analysis

OCT central B-scans, with a fixed size of 480 × 500 pixels, an estimated axial (vertical) resolution of 4.5 µm/pixel, and a lateral (horizontal) resolution of 12.5 µm/pixel for each measurement from each subject were exported for further analysis.

**Figure 2:**
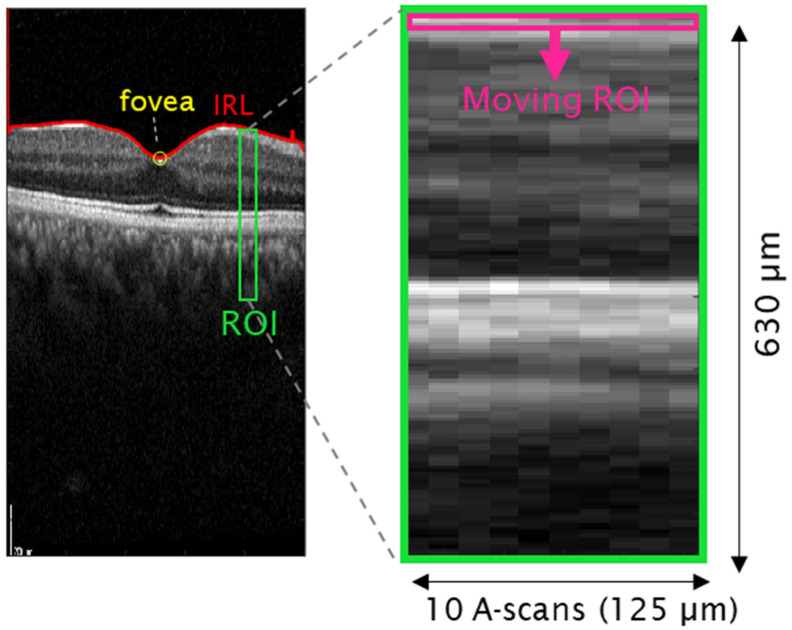
Methodology for speckle contrast estimation from each B-scan. First, image segmentation allows the demarcation of the inner retinal layer border (IRL, red line). Further, the fovea was sought as the minimum of the IRL (yellow circle). The ROI of fixed dimensions 140 × 10 pixels (green rectangle) was approximately set 1.5 mm nasally from the fovea. Inside the ROI a moving ROI of fixed dimensions 1 × 10 pixels and a moving step of 1 pixel was set (pink rectangle). The pink arrow indicates the continuation of the process.

The method of data analysis consisted of three main steps: (1) IRL segmentation and fovea location, (2) speckle contrast estimation, and (3) layer thickness evaluation. The process of IRL segmentation consists of various steps. First, median filtering was applied as a smoothing technique to eliminate noise from the image. Border detection using Canny edge detection was then implemented. After border detection (red line in Figure 2), fovea was located as the minimum value of the detected IRL border (yellow circle in Figure 2). The region of interest (ROI) was fixed on the border between parafoveal and perifoveal regions (Figure 1C); this is approximately 1.5 mm away from the macula nasally, with a fixed dimension of 140 × 10 pixels (green rectangle in Figure 2). The axial dimension of the ROI was set to 140 pixels from the IRL border. This corresponds to 630 µm approximately, which includes the whole retina and part of the choroid. The lateral dimension of the ROI was fixed to 10 A-scans (i.e., 10 pixels). After ROI selection, a moving ROI was defined within the main ROI. The moving ROI had fixed dimensions of 1 × 10 pixels, and a moving step of 1 pixel (pink rectangle in Figure 2).

The second step of data analysis consisted of estimating speckle contrast in each moving ROI. Speckle contrast, also known as contrast ratio, is a standard metric for speckle characterization. Speckle contrast is defined as the ratio between the standard deviation of pixel intensity and the mean pixel intensity of the sample [22]. Speckle contrast was estimated for each position of the moving ROI; this translates to 140 data points per eye.

Finally, to complete the data analysis, it was necessary to obtain the thickness of the IRL, ORL, and choroid [13]. As this was a retrospective study, data corresponding to IRL, ORL, and choroid thickness was taken from previous work [13] in which the Spectralis OCT built-in retinal layer segmentation software was used, following the criteria indicated in the previous section. No custom-made image processing in layer thickness estimation was applied. From the available data on IRL, ORL, and choroid thickness (in µm), the thickness of each layer in pixels was estimated. In this manner, it was possible to evaluate the mean speckle contrast corresponding to each layer and each participant.

### 2.3. Statistical Analysis

The statistical analysis was performed using Microsoft Office Excel (Microsoft Office Professional Plus 2016; Microsoft; Redmond, WA, USA). The normality of each dataset was not rejected (Shapiro–Wilk test, *p* > 0.05). The paired two-sample *t*-test and Pearson’s coefficient (r) were used to investigate both groups’ differences between right and left eyes. Group means (DM1 vs. control eyes) were considered separately for the right and left eyes. Additionally, an independent *t*-test was applied to assess speckle contrast differences between DM1 and control eyes for the different layers under analysis (IRL, ORL, and choroid). The level of significance was set to 0.05.

## 3. Results

A dataset of 148 eyes was analyzed. The dataset included 42 DM1 patients without DR and 32 controls. In particular, 84 eyes of DM1 patients (46.9% female, 53.1% male) with a mean evolution of DM1 without DR of 25.9 ± 8.4 years, and with a mean HbA1c of 7.8 ± 1.1% were included. Likewise, 64 eyes of control patients (50.0% female, 50.0% male) were included. In addition to gender, both groups were balanced in age and biometry parameters, as indicated in Table 1.

**Figure 3:**
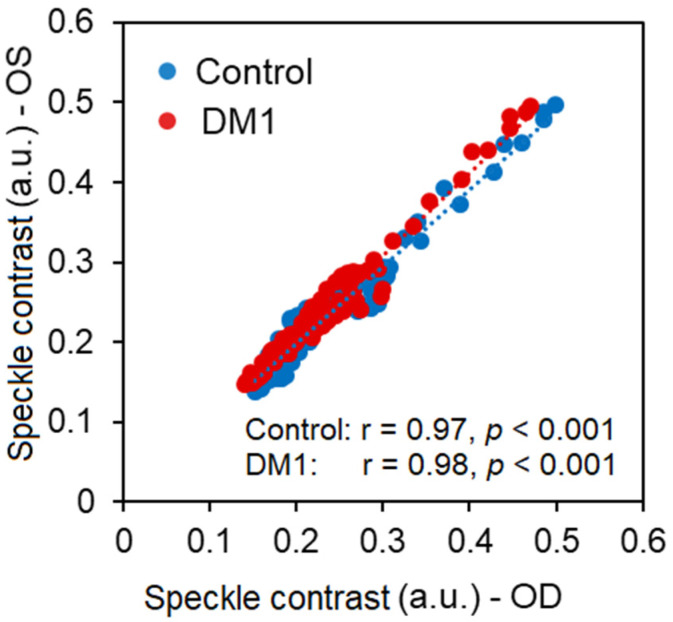
Correlation in speckle contrast between right (OD) and left eyes (OS) for control group (in blue) and DM1 group (in red). Represented data points include inner retinal layer (IRL), outer retinal layer (ORL), and choroid.

No statistically significant difference was found between left (OS) and right eyes (OD) in contrast to speckle estimation for any of the groups under analysis (OD vs. OS (paired t-test): DM1 *p* = 0.88, and control *p* = 0.78). Similarly, a strong correlation was found between left and right eyes in speckle contrast for both groups (DM1 r = 0.97 and control r = 0.98, both *p* < 0.001, Figure 3). Consequently, for simplicity, results are shown for the right eyes only, unless otherwise stated.

The analysis of layer thickness revealed that IRL was statistically significantly thicker in DM1 patients in comparison with the control group (independent *t*-test, *p* = 0.001), while no statistically significant differences were found in ORL or choroid, as indicated by Table 2.

**Figure 4:**
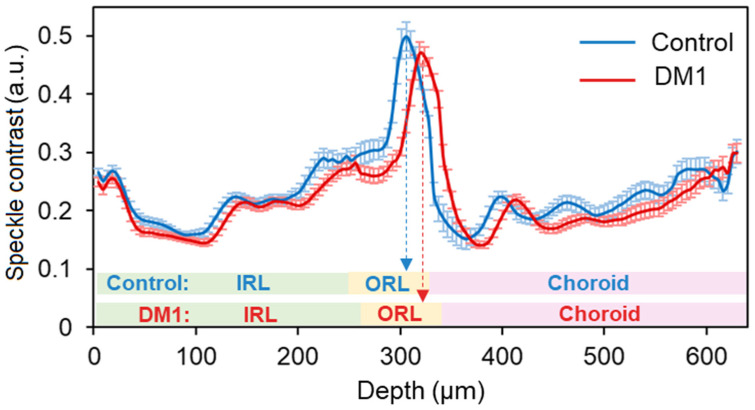
Mean speckle contrast expressed in arbitrary units (a.u.) for the control group (*n* = 32), in blue, and for the DM1 group (*n* = 42), in red. Error bars represent standard error. In the bottom part of the plot mean layer thickness per group is shown. Dashed arrows indicate the maximum speckle contrast position per group.

The analysis of speckle contrast per layer indicated that there exists a statistically significant difference in speckle contrast in the IRL layer between DM1 patients and the control group (independent *t*-test, *p* = 0.02), while no statistically significant differences were found in speckle contrast in ORL or choroid between groups, as indicated by Table 3. In addition, Figure 4 illustrates how speckle contrast evolves with depth.

Even though no statistically significant difference was found in speckle contrast in ORL between DM1 and control groups (independent t-test, *p* = 0.15), there is a statistically significant difference between groups in the maximum speckle contrast location (independent t-test, *p* = 0.008). In the control group, the maximum speckle contrast occurs in ORL depth of 58 ± 9 µm, while in the DM1 group the maximum speckle contrast occurs in a deeper position, closer to the choroid, at 64 ± 8 µm. This statistically significant difference in peak location can be observed in Figure 4.

## 4. Discussion

It has been widely proven in the literature that the image speckle is closely related to the biological structure of the tissue under study [22]. Among the many different ways to characterize the speckle of an image, the speckle contrast is the mathematically most straightforward and, consequently, the most widely used metric. In this context, the results of this study suggest that there are retinal structural differences between the patients diagnosed with DM1, compared to the control group. A difference in speckle contrast between groups indicates a tissue difference between groups, even though such differences might not be yet clinically diagnosable.

In general, OCT has become an essential tool for the retinal and choroidal study in patients with DM in its early stages and to monitor the changes that occur during the disease progression [31]. In some studies, in diabetic patients using OCT, there seemed to be retinal thickening as an early sign of DR, without DME [32,33]. In another study [13], the average total retinal thickness was assessed in the nine areas of the ETDRS, finding it thicker in patients with DM1 than in the control group, measured both with SD-OCT Spectralis and with SS-OCT Triton. In the previous study in which the different retinal layer thickness of the DM1 patients used for this study were analyzed [13], it was also interpreted that in the DM1 group the GCL, belonging to the IRL, is vulnerable to progressive damage before the appearance of microvascular DR signs. On the other hand, some studies have found that both DM1 and DM2 subjects with moderate or severe DR had a thinner GCL than subjects without DR [34,35].

Patients with DM1 without DR present thickness changes in the IRL detectable in the nine ETDRS areas. Especially in the ganglion cell bodies located in the GCL at the parafoveal level, where the N3 area studied in this work is located. These changes in thickness in the GCL are also reflected in the RNFL, formed by the ganglion cell axons, and easily measured in the perifoveal areas due to their greater thickness. These changes in the IRL, both in the RNFL and in the GCL, suggest that the beginning of retinal neurodegeneration could be a possible biomarker for disease progression and that it could even help guide treatment by serving as a biomarker for treatment efficacy, monitoring patients with DM, trying to slow down the development of DR [13].

In this study, statistically significant differences were seen between groups in the IRL thickness of the N3 area belonging to the parafoveal ring where the GCL is located, it was thicker in diabetic patients. In contrast, there were no differences in the ORL thickness between controls and DM1 patients in the N3 area or the choroid. The increased thickness in the macular area of DM patients has been described as an early DR sign, different from DME since patients with suspected DME were ruled out in these studies [31,32], and could be related to changes in the vessel permeability (in superficial capillary plexus located at the level of the RNFL) [33,36,37] or modifications in the Müller cells [33,38].

In the present study, the IRL speckle contrast was statistically significantly lower in the DM1 group without DR, while the maximum speckle contrast is reached within the ORL thickness at a deeper position, closer to the choroid in this group (Figure 4). These results reinforce the theory that the retinal tissue is affected in those patients with diagnosed DM1, although there is still no ocular pathology associated with the disease. This may be due to several factors that include preliminary neurodegeneration, undetectable vascular changes with extravasation that generate a slight retinal thickening that is also manifested in a decrease in speckle contrast due to fluid accumulation, or modifications in the size of glial cells, generating retinal thickening that is also manifested in the speckle contrast and delaying its maximum in the ORL [31,32,33,36,37,38]. The differences observed are currently of no clinical importance. Still, they suggest an alteration in the retinal tissue of DM1 patients, especially at the IRL level, so close follow-up of patients with this pathology would be recommended. Therefore, this finding (decreased speckle contrast) could support the IRL neurodegeneration before DR signs are observed.

In addition, it has been seen throughout the literature review that not only structural changes occur but also functional changes. It has been found that in patients with DR in DM1 and DM2, there are alterations in visual function with a detectable decrease in contrast sensitivity in DM1 and DM2 without DR, with greater differences when increasing the spatial frequency [39,40,41]. Numerous studies have detected the presence of color vision defects in patients with DM1 and DM2 with DR in the blue-yellow axis [42,43,44], which suggests that it may be an early manifestation of neuronal dysfunction in diabetes, with cone effects (located in the ORL) in the absence of visible microvascular changes [45,46]. On the other hand, it should be noted that the ORL remains without statistically significant differences between groups in terms of thickness and speckle contrast, showing more resistance of cones and rods to the initial structural neurodegeneration that appears in the IRL. However, it is observed that the maximum speckle contrast in DM patients occurs significantly deeper in the ORL, closer to the choroid. This indicates a tissue difference in ORL layers between groups, which could be interpreted as the beginning of damage in the ORL layers closest to the IRL, with damage starting in the innermost layers of the retina and progressing to the outermost layers. So, there is still no agreement on whether this functional impairment begins before DR; if so, this fact would also support an early degeneration of retinal neurons.

This study does not present substantial limitations. Even though the analysis area is small, it was chosen following previous literature that indicated this region as the most sensitive to suffering first retinal damage associated with DM1. Nevertheless, there is no technical limitation to further expanding the analysis area. Likewise, it would be interesting to study speckle contrast as a tissue integrity biomarker in different diabetes types and DR stages.

## 5. Conclusions

In conclusion, statistically significant differences were found in speckle contrast between the control and the DM1 without DR group, finding a more attenuated contrast in diabetes. The IRL thickness was significantly thicker in the DM1 without DR than in the control group. In addition, the maximum speckle contrast was reached in the ORL thickness for both groups, although in the DM1 group was reached closer to the choroid.

## Figures and Tables

**Table 1:** Group means, standard deviation and range of age, axial length (AL), and refraction (Rx). Independent *t*-test was applied for comparison between DM1 (*n* = 42) and control (*n* = 32) groups.

	DM1 (*n* = 42)	Control (*n* = 32)	*p*-Value
Age (years)	38.95 ± 12.50 (22, 65)	36.38 ± 10.02 (21, 62)	0.33
AL (mm)	23.59 ± 1.16 (20.82, 27.71)	23.24 ± 0.96 (20.66, 25,01)	0.15
Rx (D)	−1.03 ± 2.35 (−5.00, +5.00)	−0.94 ± 1.83 (−5.00, +4.50)	0.24

**Table 2:** Mean layer thickness of inner retinal layer (IRL), outer retinal layer (ORL), and choroid. Standard deviation and range are also indicated. Independent *t*-test was applied for comparison between DM1 (*n* = 42) and control (*n* = 32) groups.

	DM1 (*n* = 42)	Control (*n* = 32)	*p*-Value
IRL (µm)	262 ± 18 (219, 319)	254 ± 15 (228, 285)	0.001 *
ORL (µm)	82 ± 2 (76, 92)	82 ± 2 (76, 87)	0.32
Choroid (µm)	309 ± 73 (104, 475)	298 ± 83 (159, 532)	0.14

* Asterisk (*) indicates a statistically significant difference.

**Table 3:** Mean speckle contrast of inner retinal layer (IRL), outer retinal layer (ORL), and choroid, expressed in arbitrary units (a.u.). Standard deviation and range are also indicated. Independent t-test was applied for comparison between DM1 (*n* = 42) and control (*n* = 32) groups.

	DM1 (*n* = 42)	Control (*n* = 32)	*p*-Value
IRL (a.u.)	0.20 ± 0.03 (0.13, 0.26)	0.22 ± 0.03 (0.17, 0.31)	0.02 *
ORL (a.u.)	0.34 ± 0.07 (0.19, 0.51)	0.36 ± 0.06 (0.29, 0.54)	0.15
Choroid (a.u.)	0.19 ± 0.04 (0.13, 0.28)	0.21 ± 0.05 (0.13, 0.30)	0.19

* Asterisk (*) indicates a statistically significant difference.

## Data Availability

The data sets of the current study are available from the corresponding author upon reasonable request.
